# Preventive Photobiomodulation for Chemotherapy-Induced Oral Mucositis: A Systematic Review of Randomized Clinical Trials

**DOI:** 10.3390/biomedicines13020268

**Published:** 2025-01-22

**Authors:** Susell Parra-Rojas, Rocío Trinidad Velázquez-Cayón, Maria Elena Ciortan-Pop, Manoela Domingues Martins, Juliana Cassol Spanemberg

**Affiliations:** 1 Oral Medicine and Phototherapy Research Group—OMEP, Faculty of Health Sciences, Department of Dentistry, Fernando Pessoa Canary Islands University, 35450 Gran Canaria, Spain; sparra@ufpcanarias.es (S.P.-R.); rvelazquez@ufpcanarias.es (R.T.V.-C.); 2 Faculty of Health Sciences, Department of Dentistry, Fernando Pessoa Canary Islands University, 35450 Gran Canaria, Spain; meciortan@gmail.com; 3 Departament of Oral Pathology, Universidade Federal do Rio Grande do Sul, Porto Alegre 90010-150, Brazil; manomartins@gmail.com

**Keywords:** oral mucositis, photobiomodulation, chemotherapy, radiotherapy, preventive protocols

## Abstract

**Background/Objectives:** Oral mucositis (OM) is the most common acute complication among cancer patients. It initially manifests as an inflammatory process, beginning with erythema and edema of the oral mucosa, progressing to erosive lesions, and ultimately leading to highly painful ulcers. This systematic review seeks to evaluate the efficacy of preventive PBM protocols in mitigating chemotherapy-induced OM. **Methods:** The PRISMA guidelines were followed. The search was conducted in August 2024 in the following databases: Pubmed/Medline, Scopus, WoS, Cochrane, SciELO, BDTD, and BVS/IBECS. Only randomized clinical trials that utilized preventive photobiomodulation protocols in chemotherapy patients were included. All studies involving patients previously treated with radiation therapy were excluded. The Joanna Briggs Institute tool was employed for risk of bias analysis. **Results:** The total sample size consisted of 828 patients aged between 1 and 84 years. There was no predisposition based on gender or age. When the patients were evaluated under preventive protocols, some cases of mucositis manifested in a total of 339 cases. Of the total number of patients in the 13 selected studies (*n* = 828), 40.94% developed oral mucositis over the course of chemotherapy cycles. Comparing the experimental and control groups, 211 patients who did not receive preventive laser treatment developed oral mucositis; in contrast, only 128 in the experimental group did. Eighty-five percent of the studies exhibited a low risk of bias. **Conclusions:** Preventively applied photobiomodulation proves effective in minimizing or even preventing the manifestation of oral mucositis and reducing the severity of lesions that arise during oncological treatment. Registration PROSPERO (CRD42023465329).

## 1. Introduction

Oral mucositis (OM) is the most common acute complication among cancer patients undergoing chemotherapy and radiotherapy, affecting over 60% of patients and up to 85% in those receiving bone marrow transplantation. Its high prevalence and significant impact on patient care make OM a critical concern for healthcare providers [1,2,3,4,5,6,7].

Oral mucositis exhibits various manifestations, often exacerbated by the different types of oncology treatments that may be administered concurrently to each patient, depending on the type of tumor they have [3]. The clinical signs of OM range from superficial sore erythema to complete mucosal ulceration. Patients who develop OM during cancer therapy are more likely to interrupt or postpone the treatment because they could present pain, difficulty in feeding, malnutrition, dehydration, and dysgeusia, leading to a profound deterioration in quality of life. These symptoms impair oral hygiene and nutrition and cause additional trauma during chewing [1,2,3,4,5].

Preventing oral mucositis (OM) is crucial in oncology care, as its occurrence is linked to worse clinical outcomes, diminished quality of life, and increased financial burden compared to patients without such lesions [3,6]. The MASCC guidelines emphasize the importance of maintaining thorough oral hygiene and adhering to a structured prophylactic regimen. These recommendations include dietary adjustments to soft foods, the use of agents such as honey, aloe vera, glutamine, amifostine, cryotherapy, granulocyte-colony stimulating factor, keratinocyte growth factor, antibiotic pastilles or pastes, and sucralfate, along with anesthetics and palliative rinses [1,4,5,7].

Among these recommendations from MASCC, photobiomodulation (PBM) has emerged as a promising and effective strategy for both preventing and treating OM [3,4,6,8,9,10]. This therapy employs red or infrared light to deliver beneficial effects on cells and tissues, offering pain relief, anti-inflammatory benefits, and accelerated tissue healing through enhanced revascularization [11,12,13].

Extensive research underscores PBM’s efficacy as a first-line option in preventing and treating OM, with numerous studies demonstrating its ability to improve patient outcomes without causing adverse effects on the treated tissues [6,10,14,15,16,17,18]. Consequently, implementing preventive laser therapy protocols is essential for all oncology patients to mitigate the risk and severity of mucositis. In patients without visible lesions, PBM stimulates healthy tissues, reducing the risk or severity of OM. Wide-ranging research supports PBM as a safe, non-invasive, and efficient approach that enhances the quality of life for oncology patients [8,9,10,11,12,13,14,15,16,17,18,19].

Given the substantial burden of OM on cancer patients, this systematic review seeks to evaluate the efficacy of preventive PBM protocols in mitigating chemotherapy-induced OM. By validating these protocols through randomized clinical trials, we aim to advance patient care and improve outcomes in oncology settings.

## 2. Materials and Methods

The following study follows the protocols established by PRISMA (Preferred Reporting Items for Systematic Review and Meta-Analysis) [20]. The systematic review is registered in PROSPERO (International Prospective Register of Systematic Reviews) with the registration number CRD42023465329.

### 2.1. Eligibility Criteria

The articles were selected without any restrictions or limitations regarding the year of publication or languages. Only randomized clinical trials (RCTs) that used prevention protocols to avoid the development or reduce the severity of OM were chosen. Cancer patients with indications for chemotherapy treatment were included without age restrictions. Randomized clinical trials (RCTs) with at least one experimental group receiving a prophylactic protocol with PBM were included. Cancer patients with indications for chemotherapy treatment were included without age restrictions. RCTs with at least one experimental group receiving a prophylactic protocol with PBM were included. Cancer patients with prior radiotherapy (RT) or those receiving concurrent chemotherapy (CT) were excluded, as were in vitro studies, non-controlled studies, narrative reviews, systematic reviews, meta-analysis, books, editorials, editor letters, clinical manuals, and case reports. Studies where the experimental group received protocols with LED (light-emitting diode) devices were also excluded.

### 2.2. Information Sources

A systematic review of the scientific literature was conducted, searching for studies involving cancer patients that utilized preventive photobiomodulation protocols before initiating oncological treatment with the aim of preventing oral mucositis. The final search was conducted on 5 August 2024 in the following databases: Pubmed/Medline (United States National Library of Medicine), Scopus, WOS (Web of Science), Scielo (Scientific Electronic Library Online), BDTD (Biblioteca Digital Brasileira de Teses e Dissertações), BVS/IBECS (Biblioteca Virtual en Salud/Índice Bibliográfico Español en Ciencias de la Salud), and the Cochrane Library (Cochrane Database of Systematic Reviews, Cochrane Central Register of Controlled Trials-CENTRAL).

### 2.3. Search Strategy

The research question was designed based on PICO (Population, Intervention, Comparison, and Outcome) items and was used to define and guide the literature search, with the aim of obtaining results aligned with the research question. The descriptors were selected from the Medical Subject Headings (MeSH) terms and combined through Boolean operators.

The search strategy was adapted to the various databases consulted (please see the Supplementary Materials for the complete search strategy): (Photobiomodulation Therapy OR Phototherapy OR Photobiomodulation OR Low-Level Light Therapy OR Laser Phototherapy OR Low-Level Laser Therapy OR LLLT OR Low-Power Laser Irradiation OR Low-Power Laser Therapy OR Laser Irradiation OR Laser Therapies OR Low Power Laser Irradiation OR Low Power Laser Therapy OR Low-Level Laser Therapies OR Low-Level Light Therapies OR Low-Power Laser Therapies OR Photobiomodulation Therapies OR Laser Biostimulation) AND (Mucositis OR Oral mucositis OR Chemotherapy-induced Oral Mucositis OR Chemotherapy-induced Mucositis OR stomatitis OR oral mucosa inflammation) AND (Cancer Chemotherapy OR Chemotherapy).

### 2.4. Selection Process

A manual search was conducted on Google Scholar (limited to the first 100 articles), as gray literature and the references of the included articles were manually examined for potentially relevant bibliography. The articles were selected based on title according to the eligibility criteria described above. Subsequently, potentially eligible studies were included in the second phase for abstract reading, and then, in a third phase, for full-text reading. The records obtained from the databases after applying the search strategy were exported to the Zotero^®^ reference manager (version 6.0.26). All duplicate articles were removed using the same reference manager. A manual search was also conducted to inspect possible existing duplicates.

### 2.5. Data Collection Process and Data Items

A qualitative synthesis of the studies included in the review was conducted. Tables were created to summarize the information, based on the patient profile, the severity of oral mucositis, the application of extraoral and intraoral laser (prior to or during chemotherapy treatment), the dosimetry applied to each patient, and the frequency of application.

After database searches, the initial screening involved reviewing titles and abstracts, with subsequent full-text reading for the final selection. Inclusion and exclusion criteria were consistently applied. The primary study selection involved two independent reviewers who carried out an initial screening based on the review of titles and abstracts (S.P.-R and M.E.C.-P), with a third reviewer (J.C.S) resolving discrepancies. The Kappa concordance test yielded a coefficient of 0.80, indicating considerable agreement and reinforcing the reliability of the study selection process.

### 2.6. Study Risk of Bias Assessment

To assess the methodological quality of the studies included in this systematic review, the Joanna Briggs Institute (JBI) tool for randomized clinical trials was used. This tool contains 13 questions that evaluate different aspects of the internal and external validity of the studies, including selection bias, intervention administration, outcome measurement, and participant retention. Each domain was independently evaluated by two reviewers, and discrepancies were resolved by a third reviewer. The questions addressed aspects such as the appropriate randomization of participants, allocation concealment, blinding of outcome assessors, and data integrity in follow-up. Responses were categorized as “Yes”, “No”, or “Unclear”.

## 3. Results

### 3.1. Study Selection

**Figure 1:**
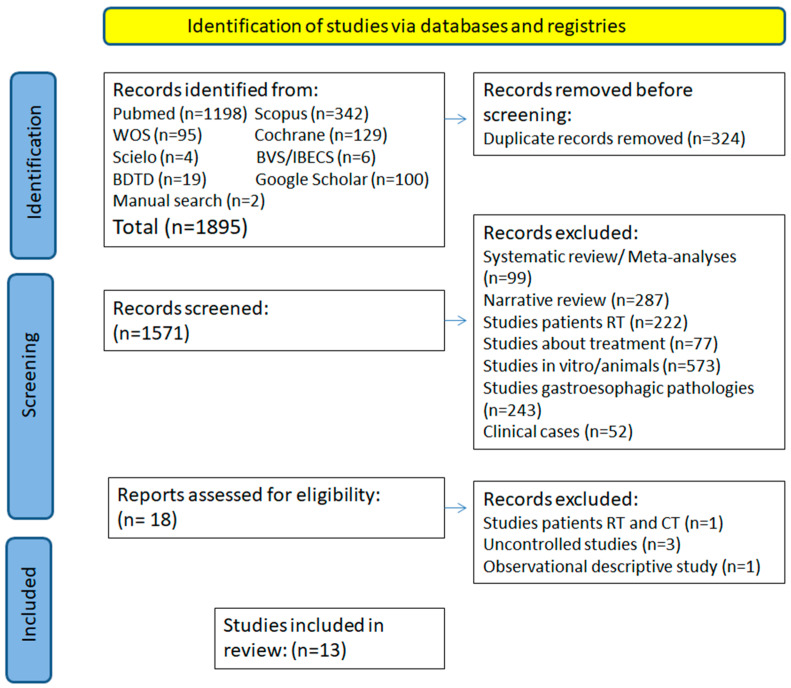
The flowchart of the included studies.

At the end of the search strategy, 1895 documents were found without applying any filters; of which 1198 were from Pubmed/Medline, 342 from Scopus, 95 from WoS, 4 from SciELO, 19 from BDTD, and 6 articles from BVS/IBECS. When necessary, in case of doubts with data or potential yet unavailable articles, the authors were contacted by email to correctly apply the inclusion and exclusion criteria. All articles that did not meet the criteria were excluded. The majority of articles were discarded as they involved studies with animals, systematic reviews/meta-analyses, clinical cases, articles with a histological focus, and those where the described oral mucositis was caused by radiotherapy and radiochemotherapy. Following this process, a total of 13 studies were included in the systematic review (Figure 1).

### 3.2. Risk of Bias in Studies

The thirteen studies included in this systematic review present a low risk of bias, according to the evaluation based on key domains such as randomization, blinding, and handling of incomplete data. The bias risk analysis was conducted using the Joanna Briggs Institute’s Critical Appraisal Tool (JBI). Out of the 13 studies assessed, 85% showed a low risk of bias (*n* = 11), with only 2 studies presenting a medium risk due to a lack of methodology data (Table 1).

### 3.3. Results of Individual Studies

The total sample size consisted of 828 patients aged between 1 and 84 years. There was no predisposition based on gender or age. Both the sample characteristics and laser usage parameters are described in (Table 2). When the patients were evaluated under preventive protocols, some cases of mucositis manifested in a total of 339 cases. Of the total number of patients in the 13 selected studies (*n* = 828), 40.94% developed oral mucositis over the course of chemotherapy cycles. Comparing the experimental and control groups, 211 patients who did not receive preventive laser treatment developed oral mucositis; in contrast, only 128 in the experimental group. Specific data, such as the OM assessment scale applied, incidence by group, and severity can be observed in (Table 3).

## 4. Discussion

In the literature, numerous studies confirm the efficacy of photobiomodulation in treating oral mucositis, yet few have rigorously established protocols for its preventive application and preparation of patients prior to chemotherapy cycles. Recognizing the global significance of oral mucositis, the MASCC/ISOO clinical guidelines recommend the prophylactic and therapeutic use of PBM [15,16]. This review focuses on RCTs utilizing photobiomodulation solemnly as a preventive measure.

OM impacts over 50% of patients, typically manifesting 3 to 7 days post-initiation of chemotherapy. High-risk drugs include 5-Fluorouracil, Adriamycin, Cyclophosphamide, Methotrexate, Etoposide, Bleomycin, Cisplatin, Vincristine, Vinblastine, and Doxorubicin, among others [14]. Currently, no antineoplastic treatment selectively targets tumor cells, inevitably causing adverse effects such as OM.

The studies included in this review encompass a total sample size of 828 patients, which is limited given OM’s prevalence among chemotherapy patients. These studies employed assessment tools such as the World Health Organization (WHO), Oral Mucositis Assessment Scale (OMAS), and the National Cancer Institute Common Terminology Criteria for Adverse Events (NCI–CTCAE) [12,13,14,15,16,17,18,19,20,21,22,23,24,25,26,27,28,29,30]. Participants, both pediatric and adult, presented with various cancers, including leukemia, head and neck cancer, and breast cancer [12,13,14,15,16,17,18,19,20,21,22,23,24,25,26,27,28,29,30]. Systemic and local factors, such as individual immune responses and oral health can vary (children requiring adult supervision) and influence OM susceptibility. Factors like oral biofilm accumulation can exacerbate microbiota imbalances, increasing the likelihood of OM development. Furthermore, it should be noted that multiple factors can interfere with the response to PBM since each patient is unique, and not all patients respond in the same way.

The mechanisms of action of PBM involve red and infrared spectra triggering distinct stimuli. Red-spectrum radiation stimulates mitochondrial biostimulation, while infrared wavelengths affect plasma membrane channels. Both spectra initiate biochemical cascades post-photoreaction [10,11]. PBM generates no heat in the treated area, with its core properties including analgesia, anti-inflammatory effects, and biostimulation. These effects stem from light absorption by chromophores, primarily cytochrome C oxidase and porphyrins, which enhance cellular metabolism through ATP production, protein synthesis, oxidative respiration, and increased oxygen consumption [10].

Beyond its proven efficacy in preventing oral mucositis, the prophylactic use of photobiomodulation offers promising therapeutic potential for a range of other oral pathologies. PBM’s capacity to enhance tissue regeneration and mitigate inflammation suggests its application may extend to conditions such as xerostomia, oral lichen planus, recurrent aphthous stomatitis, pemphigus vulgaris, herpes labialis, bisphosphonate-induced osteonecrosis of the jaw, and trigeminal neuralgia, among others [9,11]. When compared to conventional therapies for oral mucositis—such as cryotherapy, 0.12% chlorhexidine, topical anesthetics, 0.03% triclosan rinses, analgesics, sucralfate, and other pharmacological interventions [7], PBM distinguishes itself through its non-invasive approach, minimal adverse effects, and capacity to accelerate healing across diverse oral tissues.

A particularly noteworthy advantage of PBM lies in its potential to significantly reduce reliance on analgesic medications for pain management. By alleviating pain through carefully optimized PBM protocols, the need for anti-inflammatory and analgesic drugs may be diminished, thereby reducing medication-related side effects and improving overall patient outcomes. Although these benefits underscore PBM’s promise, additional robust studies are imperative to fully elucidate its efficacy across various oral conditions and to enable direct comparisons with alternative therapeutic modalities.

No universally accepted PBM protocol exists for OM prevention or treatment. Recommendations range from initiating therapy during the first chemotherapy cycle to beginning preventive protocols 10 days prior. Application frequency and methods (intraoral, extraoral, or combined) vary, with some advocating daily or weekly treatments [8,31,32,33,34,35,36,37,38,39,40,41].

Menezes et al. [25] reported OM incidence at 14.7% in PBM-treated patients compared to 69.9% in untreated patients. Pires-Marques et al. [31] demonstrated accelerated healing with intraoral PBM applied four times weekly, reducing lesion resolution time to 11 days. Arbabi-Kalati et al. [23] highlighted the effectiveness of a 630 nm wavelength laser, with OM affecting only 58% of the laser-treated group versus 100% in controls, where severe grade 3 OM was exclusive to the latter. Gautam et al. [32] observed improved nutrition and reduced opioid use among PBM-treated patients, with no chemotherapy interruptions. Eduardo et al. [36] confirmed PBM’s safety and efficacy in pediatric oncology, particularly with supportive oral care [33,34,35,36,37,38].

Most studies employed red lasers (630–660 nm) for OM prevention and treatment, with some using infrared lasers (830–940 nm). The dose varied widely, from 2 to 80 J/cm² [21,22,23,24,25,26,27,28,29,30]. De Castro et al. [26] used both red and infrared wavelengths, finding greater efficacy with red lasers [42,43,44,45,46,47,48,49,50,51,52]. Site-specific application (intraoral or extraoral) has proven safe and effective, with extraoral methods beneficial for uncooperative pediatric patients or those with restricted oral cavity access [30]. Preventive protocols often begin with the first chemotherapy session, with applications ranging from daily to every other day. Stocker et al. [50] applied PBM daily from chemotherapy onset to patient discharge.

Wong et al. [51] instructed patients in oral hygiene before antineoplastic treatment, noting that only 3 out of 15 developed OM when PBM was initiated pre-treatment. PBM consistently reduced OM incidence and severity across trials. However, Cruz et al. [24] found no statistically significant benefit in pediatric patients predisposed to OM from prior chemotherapy cycles. Factors such as deteriorated nutritional status and poor oral hygiene, often reliant on caregivers, complicate outcomes in such cases.

PBM could yield excellent results if used preventively, ideally starting at least 1 week before the first chemotherapy session, with an optimal period of 10–14 days in advance. This aims to protect and prepare tissues for the toxicity of the CT treatment they will undergo. It is crucial to establish oral hygiene and care protocols in oncology services. Intensive oral care is paramount during all stages of treatment and will help prevent many adverse effects in patients, providing them with a better quality of life related to oral health. The dentist is the most qualified professional to assess and treat oral diseases, as well as to implement preventive measures for the ongoing oral health of cancer patients.

## 5. Limitations

There is no consensus regarding the preventive and therapeutic protocols of laser therapy, and the 13 identified studies used different dosages, durations, power levels, and wavelengths, obtaining diverse results without a standardized pattern for reference. Another significant limitation is the scarcity of randomized controlled trials specifically investigating prevention protocols implemented 10–14 days before the initiation of chemotherapy sessions. The studies were conducted with a limited number of patients, and there is a lack of descriptive data regarding their general health and lifestyle (dietary habits, stress, toxic habits such as smoking and alcohol consumption, etc.) that could be correlated with oral mucositis. Despite these limitations, this review has been able to emphasize the importance of caring for oncology patients at all stages of their treatment, aiming to prevent or treat common side effects such as oral mucositis using photobiomodulation.

## 6. Conclusions

The results indicate that prophylactic photobiomodulation is effective in reducing the development and severity of oral mucositis in patients undergoing chemotherapy. Red laser has been more commonly used in intraoral applications, while infrared has been more common in extraoral applications. The diversity of protocols studied by different research groups makes it challenging to analyze the ideal dosage. Despite variability among the reviewed studies, PBM is considered safe, non-invasive, and without side effects. There is a recognized need for more RCTs with well-established methodological criteria on this topic. The influence of different laser parameters and PBM programs underscores the importance of determining the optimal configuration for PBM, which should be the focus of future studies.

## Figures and Tables

**Table 1:** Risk of bias in studies.

Critical Appraisal Checklist for Randomized Controlled Trials	Rozza de Menezes et al. [21]	Silva et al. [17]	Salvador et al. [22]	Arbabi-Kalati et al. [23]	Cruz et al. [24]	Menezes et al. [25]	De Castro et al. [26]	Guimarães et al. [27]	Abramoff et al. [14]	Boris et al. [15]	Ferreira et al. [16]	Ahmed et al. [28]	Gambirazi [29]
1. Was true randomization used for assignment of participants to treatment groups?	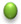	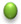	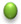	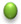	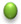	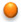	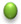	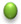	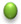	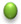	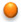	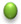	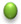
2. Was allocation to treatment groups concealed?	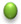	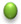	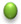	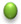	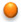	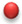	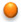	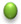	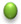	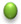	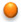	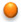	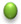
3. Were treatment groups similar at the baseline?	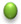	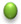	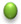	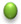	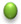	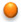	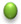	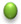	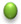	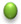	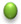	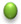	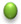
4. Were participants blind to treatment assignment?	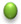	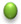	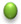	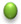	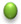	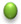	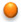	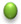	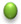	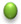	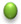	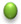	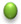
5. Were those delivering treatment blind to treatment assignment?	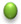	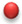	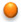	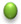	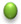	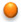	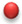	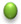	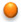	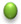	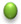	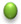	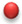
6. Were outcomes assessors blind to treatment assignment?	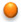	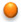	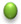	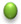	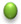	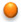	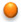	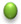	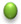	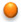	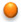	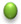	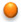
7. Were treatment groups treated identically other than the intervention of interest?	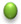	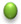	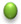	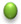	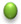	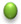	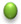	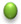	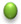	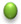	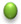	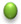	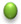
8. Was follow up complete and if not, were differences between groups in terms of their follow up adequately described and analyzed?	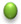	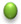	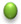	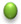	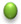	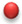	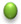	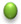	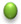	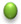	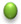	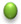	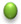
9. Were participants analyzed in the groups to which they were randomized?	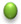	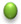	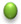	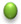	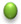	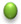	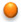	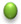	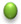	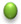	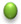	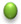	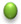
10. Were outcomes measured in the same way for treatment groups?	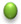	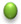	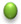	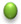	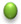	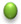	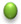	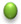	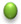	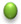	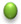	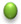	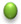
11. Were outcomes measured in a reliable way?	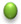	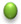	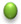	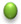	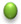	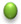	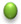	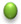	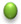	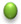	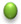	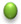	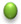
12. Was appropriate statistical analysis used?	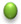	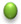	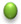	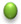	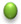	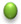	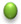	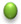	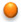	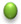	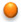	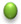	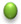
13. Was the trial design appropriate, and any deviations from the standard RCT design (individual randomization, parallel groups) accounted for in the conduct and analysis of the trial?	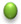	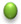	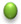	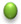	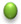	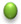	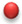	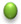	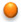	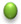	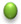	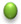	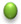
Yes 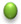 No 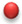 Unclear 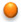													

**Table 2:** Description of the included studies.

Author	Year	Country	Condition	Age	(*n*)	Use of Laser	Wavelength (nm)	Power (nW)/ Energy Density (J/cm^2^)	Type of Laser	Application
Rozza de Menezes et al. [21]	2018	Brazil	Solid tumor	27–84 years	48 Patients	Prevention	Red 660 nm	50 mW 4 J/cm^2^	InGaAIP	Daily from CT until 3 days later
Silva et al. [17]	2014	Brazil	Hematoligical tumor	14–17 years	25 Patients	Prevention/Treatment	Red 660 nm	40 mW 4 J/cm^2^	InGaAIP	Daily from CT until 7 days later
Salvador et al. [22]	2017	Brazil	Hematological tumor	14–17 years	51 Patients	Prevention	Red 660 nm	40 mW 4 J/cm^2^	InGaAIP	Daily from CT until 7 days later
Arbabi-Kalati et al. [23]	2013	Iran	NS	18 years or more	48 Patients	Prevention	Red 630 nm	30 mW 5 J/cm^2^	NS	1 day before CT Weekly
Cruz et al. [24]	2007	Brazil	Solid tumor/hematological tumor	3–18 years	60 Patients	Prevention	Infrared 780 nm	60 mW 4 J/cm^2^	InGaAIP	Daily from CT until 5 days later
Menezes et al. [25]	2021	Brazil	Solid tumor	NS	287 Patients	Prevention/Treatment	Red 630 nm	30 mW 2 J/cm^2^	InGaAIP	Daily from CT until 7 days later
De Castro et al. [26]	2013	Brazil	Hematological tumor	1–18 years	40 Patients	Prevention/Treatment	Red 660 nm + Infrared 830 nm	100 mW 35 J/cm^2^	InGaAIP	Daily from CT until 5 days later
Guimarães et al. [27]	2021	Brazil	Hematological tumor	4–12 years	80 Patients	Prevention	Red 660 nm	100 mW 2 J/cm^2^	InGaAIP	Daily from CT until 7 days later
Abramoff et al. [14]	2007	Brazil	Hematological tumor	7–23 years	13 Patients	Prevention/Treatment	Red 685 nm	35 mW 72 J/cm^2^	AsGaAI	Alternate days from CT 7 days
Boris et al. [15]	2016	Rep. Bielorrusia	Hematological tumor	NS	33 patients	Prevention/Treatment	Red 670 nm	30 mW 21,24 J/cm^2^	InGaAIP	Alternate days from CT
Ferreira et al. [16]	2013	Brazil	Solid tumor	29–83 years	28 patients	Prevention/Treatment	Red 660 nm	40 mW 80 J/cm^2^	InGaAIP	1 day before CT and every 2 days until discharge
Ahmed et al. [28]	2015	Iraq	Solid tumor/hematological tumor	14–15 years	67 patients	Prevention/Treatment	Infrared 940 nm	0.3 mW 4.2 J/cm^2^	AIGaInAs	Daily from CT
Gambirazi [29]	2007	Brazil	Solid tumor	37–82 years	48 patients	Prevention	Red 660 nm	40 mW 3 J/cm^2^	AlGaInP	1 day before CT Weekly

NS: not specified. CT: Chemotherapy.

**Table 3:** Results regarding the incidence and severity of oral mucositis in the selected studies.

Author	(*n*)	Control Group	Incidence of OM	Severity	Scale
Rozza de Menezes et al. (2018) [21]	Exp: 12 Control: 36	Intensive oral care, chlorhexidine or triclosan mouthwash	Exp: 6 Control: 25	Exp: 3 G1, 3 G2–3 Control: 19 G1, 5 G2–3	OMAS
Silva et al. (2014) [17]	Exp: 11 Control: 14	Laser off	Exp: 8 Control: 8	Exp: 8 G1 Control: 8 G2–3	WHO
Salvador et al. (2017) [22]	Exp: 27 Control: 24	Laser off	Exp: 5 Control: 14	Exp: 5 G2 Control: 10 G2, 4 G3	WHO
Arbabi-Kalati et al. (2013) [23]	Exp: 24 Control: 24	Laser off	Exp: 10 Control: 24	Exp: 8 G1, 2 G2 Control: 2 G1, 12 G2, 10 G3	WHO
Cruz et al. (2007) [24]	Exp: 29 Control: 31	Laser off	Exp: 13 Control: 7	Exp: 6 G1, 5 G2, 2 G3 Control: 5 G1, 1 G2, 1 G3	NCI–CTCAE
Menezes et al. (2021) [25]	Exp: 204 Control: 83	Laser off	Exp: 30 Control: 48	Exp: 22 G1, 8 G2 Control: 19 G1, 30 G2, 9 G3	WHO
De Castro et al. (2013) [26]	Exp: 20 Control: 20	Laser off	Exp: 8 Control: 15	Exp: 5 G1, 3 G2 Control: 4 G1, 6 G2, 5 G3	WHO
Guimarães et al. (2021) [27]	Exp: 40 Control: 40	LED	Exp: 4 Control: 5	Exp: 1 G1, 2 G2,1 G3 Control: 1 G1,3 G2, 1 G3	WHO
Abramoff et al. (2007) [14]	Exp: 6 Control: 7	Laser off	Exp: 1 Control: 3	Exp: 1 G2 Control: 3 G1	NCI–CTCAE
Boris et al. (2016) [15]	Exp: 17 Control: 16	Oral care	Exp: 4 Control: 8	NS	WHO
Ferreira et al. (2013) [16]	Exp: 14 Control: 14	Oral care	Exp: 6 Control: 9	Exp: 1 G1, 2 G2, 1 G3, 2 G4 Control: 3 G1, 4 G2, 2 G3	WHO
Ahmed et al. (2015) [28]	Exp: 34 Control: 33	Laser off	Exp: 18 Control: 24	NS	WHO
Gambirazi (2007) [29]	Exp: 25 Control: 23	Oral care Laser off	Exp: 15 Control: 21	Exp: 10 G1–2, 5 G3–4 Control: 12 G1–2, 9 G3–4	WHO

Exp—experimental; NS—no specified; OM—oral mucositis; WHO—World Health Organization; NCI-CTCAE—Common Terminology Criteria for Adverse Events, National Cancer Institute; OMAS—Oral Mucositis Assessment Scale.

## Data Availability

The datasets generated during and/or analyzed during the current study are available from the corresponding author on reasonable request.

## Supplementary Materials

The following supporting information can be downloaded at https://www.mdpi.com/article/10.3390/biomedicines13020268/s1.
