# Supplementary material for: Preventive Photobiomodulation for Chemotherapy-Induced Oral Mucositis: A Systematic Review of Randomized Clinical Trials
Source: Biomedicines. 2025 Jan 22;13(2):268. doi: 10.3390/biomedicines13020268 (PMC11852607; doi:10.3390/biomedicines13020268)
Supplement: Supplementary file 1 [file biomedicines-13-00268-s001.zip › biomedicines-3383789-supplementary.pdf]

SEARCH STRATEGY

Pubmed/Medline:

(Photobiomodulation Therapy OR Phototherapy OR Photobiomodulation OR Low-Level Light Therapy OR Laser Phototherapy OR Low-Level Laser Therapy OR LLLT OR Low-Power Laser Irradiation OR Low-Power Laser Therapy OR Laser Irradiation OR Laser Therapies OR Low Power Laser Irradiation OR Low Power Laser Therapy OR Low-Level Laser Therapies OR Low-Level Light Therapies OR Low- Power Laser Therapies OR Photobiomodulation Therapies OR Laser Biostimulation) AND (Mucositis OR Oral mucositis OR Chemotherapy-induced Oral Mucositis OR Chemotherapy-induced Mucositis OR stomatitis OR oral mucosa inflammation) AND (Cancer Chemotherapy OR Chemotherapy)

Scopus:

Strategy – Field – Article title, abstract, keyword

Phototherapy OR Photobiomodulation OR Low-Level Light Therapy OR Laser Phototherapy OR Low-Level Laser Therapy OR LLLT AND Cancer Chemotherapy OR Chemotherapy

WOS:

Photobiomodulation therapy (Topic) and Oral mucositis (Topic) and Chemotherapy (Topic)

BVS (Biblioteca virtual en salud)/IBECS (Índice bibliográfico español en ciencias de la salud):

Título, resumen y asunto

(Photobiomodulation Therapy OR Phototherapy OR Photobiomodulation OR Low-Level Light Therapy OR Laser Phototherapy OR Low-Level Laser Therapy OR LLLT OR Low-Power Laser Irradiation OR Low-Power Laser Therapy OR Laser Irradiation OR Laser Therapies OR Low Power Laser Irradiation OR Low Power Laser Therapy OR Low-Level Laser Therapies OR Low-Level Light Therapies OR Low- Power Laser Therapies OR Photobiomodulation Therapies OR Laser Biostimulation) AND (Mucositis OR Oral mucositis OR Chemotherapy-induced Oral Mucositis OR

Chemotherapy-induced Mucositis OR stomatitis OR oral mucosa inflammation) AND (Cancer Chemotherapy OR Chemotherapy)

SciELO:

Strategy – Field – Article title, abstract, keyword

Phototherapy OR Photobiomodulation OR Low-Level Light Therapy OR Laser Phototherapy OR Low-Level Laser Therapy OR LLLT AND Cancer Chemotherapy OR Chemotherapy

BDTD:

All fields

fotobiomodulaçao OR fototerapia OR laser de baixa potencia AND mucosite oral AND quimioterapia

Cochrane:

(Photobiomodulation Therapy OR Phototherapy OR Photobiomodulation OR Low-Level Light Therapy OR Laser Phototherapy OR Low-Level Laser Therapy OR LLLT OR Low-Power Laser Irradiation OR Low-Power Laser Therapy OR Laser Irradiation OR Laser Therapies OR Low Power Laser Irradiation OR Low Power Laser Therapy OR Low-Level Laser Therapies OR Low-Level Light Therapies OR Low- Power Laser Therapies OR Photobiomodulation Therapies OR Laser Biostimulation) AND (Mucositis OR Oral mucositis OR Chemotherapy-induced Oral Mucositis OR Chemotherapy-induced Mucositis OR stomatitis OR oral mucosa inflammation) AND (Cancer Chemotherapy OR Chemotherapy)
